# Supplementary material for: Moving beyond Symptom Criteria to Diagnose and Treat Functional Disorders: Patient-Reported Symptoms of Functional Lower Gastrointestinal Disorders Correlate Poorly with Objective Assessment of Luminal Contents Seen on Intestinal Ultrasound
Source: J Clin Med. 2024 Aug 13;13(16):4759. doi: 10.3390/jcm13164759 (PMC11355646; doi:10.3390/jcm13164759)
Supplement: Supplementary file 1 [file jcm-13-04759-s001.zip › jcm-3069293-supplementary.pdf]

Supplemental material

Table S1. Symptom change correlations with change in ultrasound score

Symptom change	Spearman's Correlation Co-efficient	P value
Upper abdominal pain	-0.009	.959
Lower abdominal pain	-0.198	.924
Diarrhoea	-0.017	.924
Constipation	0.162	.352
Overall symptoms	-0.045	.802
Quality of life	-0.136	.444
